# Evoking nostalgia by presenting hit-song lists

**DOI:** 10.3389/fpsyg.2026.1800653

**Published:** 2026-05-29

**Authors:** Satoshi Kawase, Kei Eguchi

**Affiliations:** 1 Faculty of Psychology, Kobe Gakuin University, Kobe, Japan; 2 Independent Researcher, Osaka, Japan

**Keywords:** emotion, hit-song lists, Japan, nostalgia, social bond

## Abstract

**Introduction:**

This study investigates the nostalgia evoked by informational music cues, specifically lists of hit songs presented as text, without auditory presentation. The primary aim is to clarify the psychological characteristics and functions of nostalgia elicited by recognizing culturally shared music-related information, rather than examine nostalgia induced by music listening.

**Methods:**

We examined nostalgia, its function, and mood while looking at hit-song lists, by collecting large-scale data on specific ages and hit-song lists in a controlled manner. Participants between 20 and 69 years of age responded to the hit-song lists in Japan between 1983 and 2022, and a total of 3,741 responses were obtained.

**Results:**

The results showed that nostalgia was effectively evoked by simply observing the song titles and musicians’ names, and that the more songs the participants knew, the stronger the response. The strongest correlation within the nostalgia function was with self-continuity. Nostalgia peaked in the music hit lists that were associated with participants’ late adolescence, which was consistent with the reminiscence bump phenomenon. No significant relationship was observed between nostalgia and mood change.

**Discussion:**

Text-based music cues such as hit lists can function as powerful triggers for nostalgia. The present study highlighted a previously underexplored pathway through which nostalgia can be triggered in everyday contexts. Associations were observed between nostalgia and both self-continuity and social bonding, suggesting that informational musical cues may support psychological connections with oneself and others through symbolic cultural artifacts.

## 1 Introduction

Nostalgic music takes people back to the past. Nostalgia is a past-oriented emotional experience, characterized by a bittersweet affective tone, encompassing both positive and negative emotions (Sedikides and Wildschut, 2018, 2022). Recently, the positive aspects of nostalgia have attracted considerable attention. A survey of university students showed that recalling nostalgic memories evoked more positive emotions than negative emotions, such as loss (Wildschut et al., 2006). Conversely, nostalgia can negatively affect everyday life, causing homesickness (Newman et al., 2020). Accordingly, emotions evoked by nostalgic experiences are a mixture of positive and negative emotions (Holak and Havlena, 1998).

Nostalgia evokes psychological responses related to connections with others and the significance of one’s existence. A review on nostalgia pointed out that it is induced by significant others and life events involving them and can be a coping strategy for loneliness and social exclusion by activating social bonds (Sedikides and Wildschut, 2019). Although loneliness increases nostalgia, nostalgia increases the perception of social support (Zhou et al., 2008). Furthermore, nostalgia provides cultural universality. Regardless of cultural differences, nostalgia promotes help-seeking behavior by increasing social connectedness (Juhl et al., 2021). Additionally, nostalgia can help individuals cope with loneliness by increasing their sense of well-being (Sedikides et al., 2022). Therefore, nostalgia is linked to various psychological responses, such as sociality and well-being.

Considering these findings, nostalgia comprises several functional factors. These include social connectedness, meaning in life, and self-continuity (Sedikides and Wildschut, 2022). Social connectedness is the recognition that one is connected to society, such as through memories of close individuals or significant events involving other individuals. Meaning in life relates to one’s identity and is a factor related to the meaning of life, such as the value of one’s existence. Self-continuity emphasizes the connection between a person’s past and present and promotes the sense that one does not change over time. Kusumi (2021) proposed four factors in a survey conducted in Japan, adding self-clarification (i.e., recognition of one’s own identity) to the aforementioned factors. These findings suggest that nostalgia evokes emotional, social, and personal responses.

Listening to music is a significant trigger that evokes feelings of nostalgia (Krumhansl and Zupnick, 2013; Sedikides et al., 2022). Unexpected music can trigger the recall of past events, causing feelings of sadness and loneliness (Sloboda and O’Neill, 2001). Nostalgia is one of the most frequently experienced emotions during daily listening (Juslin and Laukka, 2004), and is the third most common emotion induced by music (Janata et al., 2007). Kusumi et al. (2010) found that music was the most important element for evoking feelings of nostalgia by TV advertisements.

Individual musical experiences are related to nostalgia. A key factor is familiarity with the music. Familiar music evokes more nostalgia (Barrett et al., 2010; Janata, 2009). Nostalgia evoked by listening to popular music excerpts is related to familiarity with the stimulus, its importance to individuals, arousal, and positive emotions (Barrett et al., 2010). A comparison between familiar and unfamiliar music revealed that highly familiar songs prompted autobiographical memories more frequently and rapidly while also eliciting more positive recollections (Jakubowski and Francini, 2023). This finding supports the notion that familiar songs facilitate memory retrieval and evoke related emotions. As hit songs are heard repeatedly by many people, it is reasonable to consider hit lists from the perspective of familiarity with music and nostalgia. Music-induced nostalgia can foster a sense of continuity in life (Sedikides et al., 2016). Nostalgia strengthens a person’s sense of continuity; that is, the sense that one’s past, present, and future are connected (Sedikides et al., 2016). This sense of coherence is essential for psychological health (Nygren et al., 2005). Nostalgia evoked by music gives strength to live in the present and future by looking back on the past (Routledge et al., 2011). Music-evoked nostalgia promotes feelings of social connectedness by fostering feelings of acceptance of reality and social support. Furthermore, people who experience musical nostalgia tend to show higher levels of social connectedness than those who do not (Routledge et al., 2011).

Research on music listening has increasingly examined autobiographical memory processes. In studies of music listening (Krumhansl and Zupnick, 2013), the phenomenon known as “reminiscence bump”—whereby memories from a specific period of youth are more easily recalled and regarded as personally significant—has been consistently observed (Jakubowski et al., 2020; Janssen et al., 2008; Rathbone et al., 2017). This pattern appears to be robust across cultures (Renwick and Woolhouse, 2023), although its strength may vary depending on the task (Kopiez et al., 2021).

Furthermore, a growing body of research on music-evoked autobiographical memories (MEAMs) has demonstrated that music can trigger vivid, emotionally salient, and temporally specific recollections of past events (Belfi et al., 2015; Jakubowski et al., 2023; Jakubowski et al., 2020; Jakubowski and Ghosh, 2019). These memories tend to be temporally aligned with the life period in which the music was originally experienced, suggesting a close correspondence between musical cues and specific stages of life (Tanberg et al., 2025). In addition, research on MEAMs indicates that the effectiveness of musical cues is relatively well-preserved across one’s lifespan and may remain robust even in older adults and clinical populations (Cuddy et al., 2015; Baird et al., 2018; O’Shea et al., 2025). Thus, these findings suggest that music-related cues may exhibit both age-related and enduring effects. However, it remains unclear whether responses to informational music cues without auditory input, which constitute the primary focus of the present study, exhibit systematic patterns consistent with established autobiographical memory processes. To our knowledge, particularly in the Japanese context, few studies have examined such responses across a wide age range.

Listening to music is not always a prerequisite for memory retrieval. A survey of university students found little difference in the vividness of emotions and memories evoked by various cues (song titles, music excerpts, lyrics, and album cover photos) related to past hit songs (Cady et al., 2008). Furthermore, the presentation of song titles alone can successfully trigger autobiographical memories (Jakubowski et al., 2020). These findings suggest that textual information, such as titles, can elicit memories even in the absence of auditory stimuli. Hit songs are not only listened to repeatedly by a broad audience but their titles also become deeply ingrained in memory, forming shared musical experiences within specific generations and cultures. Consequently, hit-song lists can serve as a potent cue for evoking individual memories.

This study investigates nostalgia evoked by informational music cues, specifically hit-song lists presented as text, without auditory presentation. The primary aim is not to examine nostalgia induced by music listening but to clarify the psychological characteristics and functions of nostalgia elicited by recognizing culturally shared music-related information. This study focused on the following three points: First, previous studies have predominantly focused on nostalgia triggered by listening to music. During recent daily music listening, such as on YouTube or Spotify, a song’s name is available in advance. Listeners can select whether to listen to it based on this information. Viewing this information makes the listener aware of the songs and may affect their decision-making about listening to the songs. Therefore, examining the psychological response when information about a song is presented is significant not only for understanding nostalgia from music but also for examining its function as a trigger for subsequent listening behavior (and occasionally consumption behavior).

Second, this study examined whether responses to these non-listening music cues were similar to those of previous studies in terms of nostalgia. Although many people feel nostalgia, hit songs vary by culture. That is, the songs and genres that become hits differ across cultures, leading to rich, diverse experiences in music listening. These cultural experiences occur across all ages. The fact that listening to music may trigger strong emotional responses in listeners (Cowen et al., 2020) across cultures and ages demonstrates the powerful influence of music itself on people. However, the kinds of songs that become hits and the emotions they evoke may differ. To the best of our knowledge, very few large-scale and systematic studies comprehensively cover recent hit songs in Japan. Therefore, the present study has the potential to provide a valuable cross-cultural perspective.

Third, the presentation of music information without listening to music may reflect real life but differ from emotional responses to listening to music. Considering the finding that nostalgia has functions such as social connections (Sedikides et al., 2016), this study examined whether such a function of nostalgia could also be observed in the presentation of hit-song lists. This investigation aims to clarify whether the nostalgia that arises when music is presented as textual information has the same characteristics as general nostalgia.

To address these issues, a large-scale survey was conducted based on the following research questions.

*RQ1*: Do hit-song lists presented as textual information evoke nostalgia?

*RQ2*: How does individual familiarity with the listed songs relate to the intensity and functions of nostalgia?

*RQ3*: Does nostalgia evoked by informational music cues engage psychological functions such as self-continuity and social connectedness?

## 2 Methods

### 2.1 Participants

A total of 3,741 valid responses were analyzed after excluding responses that exhibited straight-lining patterns (i.e., providing identical responses across all scale items). Participants were evenly distributed between the ages of 20 and 69 years, with minimal gender imbalance. A total of 40 participants, 20 men and women each, were assigned to each of the 100 groups by multiplying the 10 different hit-song lists by the 10 different ages. In the present study, to examine psychological tendencies at the group level in response to hit-song lists, participants were sampled to cover a wide age range from younger to older adults. This design allowed us to reduce the influence of specific age groups or particular hit-song lists on the observed results.

### 2.2 Materials

Ten hit-song lists containing Japanese hit songs [according to Oricon (Oricon Inc.); Billboard Japan] every 4 years from 1983 to 2022 were used in the experiment. The list included 4 years with a set of five hit songs for each year, with the names of the songs and artists. In most cases, each list consisted of 20 songs presented in a randomized order, with all ranking and temporal information removed (hereafter, the hit lists). However, if the same song was a hit across years, it was regarded as one song. Owing to errors by the experimenter, three songs were omitted from the list in 2019. The concept of the hit lists familiarity was operationalized as individual familiarity, defined as the proportion of songs that participants reported having heard, regardless of liking.

The survey used items such as the Japanese version of the Expanded Function of Nostalgia Scale (Kusumi, 2021), Two-Dimensional Mood Scale (Sakairi, 2022), and the Japanese version of the Goldsmiths Musical Sophistication Index (Gold-MSI) (Sadakata et al., 2023). Nostalgia was rated on a seven-point Likert scale to indicate the degree to which the respondents were nostalgic when they saw the hit lists (hereinafter referred to as the nostalgia score).

The Japanese version of the Expanded Function of Nostalgia Scale consists of 16 items with four subscales: *social connectedness,* which refers to social connectedness with others; *self-continuity,* which refers to being connected from the past to the present; *self-clarity,* which refers to knowing oneself; and *meaning in life,* which refers to whether one feels a sense of meaning and purpose in life. Each item is rated on a 6-point Likert scale (1 = not applicable at all; 6 = very applicable). The Two-Dimensional Mood Scale is an 8-item scale that measures mood. Based on the responses to each item, the respondent’s mood at that moment is expressed in two dimensions: valence and arousal. Each item is rated on a 6-point Likert scale (0 = not at all, 5 = strongly agree).

The Gold-MSI assesses respondents’ level of musical sophistication. The scale includes several factors; however, the *active engagement* factor was used in this study. This factor concerns engagement with music; that is, how much time or money is spent on music.

### 2.3 Procedure

The survey was conducted online by a research company (Cross Marketing Inc.). An outline of the study and informed consent forms were presented at the beginning of the survey. Individuals were presented with a description of the study and informed consent, and those who agreed to it proceeded to the survey. The informed consent form stated that the survey would be analyzed anonymously, participation in the survey was voluntary, participants would not be disadvantaged if they discontinued or withdrew from the survey, and the data would not be used for any purpose other than that of the study. Only those who agreed with these conditions participated in the survey.

After responding to the Japanese version of the Gold-MSI and the Two-Dimensional Mood Scale, participants were presented with a hit list. For each song on the hit lists, participants selected one of the following options: “have heard this song and like it,” “have heard it and do not like it,” or “have never heard it.” Subsequently, they responded to the Two-Dimensional Mood Scale, the Expanded Function of Nostalgia Scale, and an item on nostalgia. They also answered whether they had heard of, liked/heard of, or dislike/never heard each song on the list. This was implemented to examine the familiarity of the songs while simultaneously preventing participants from skipping through the list.

### 2.4 Analysis

Mean values were calculated for each combination of the 10 hit lists and 10 age groups, resulting in 100 aggregated cells, each consisting of approximately 40 participants. This approach was adopted to examine the interaction between participants’ age group and the historical period of the hit lists (i.e., chart year), with a particular focus on group-level patterns such as the reminiscence bump. Because individual responses were nested within specific age × song-list combinations, each cell was treated as a meaningful unit reflecting shared cultural experiences with music.

Although multilevel modeling or individual-level regression analyses are valid alternatives, the present study focused on aggregated patterns across age groups and chart periods. Therefore, analyzing cell-level averages allowed for a more direct and interpretable examination of group-level trends and their interactions. Furthermore, aggregation reduced individual-level variability, thereby clarifying systematic patterns across conditions. This approach is consistent with previous research examining age-related patterns in autobiographical memory and music-evoked nostalgia at the group level (e.g., Jakubowski et al., 2020), and is analogous to ecological or cell-level analyses commonly used in large-scale survey designs. Accordingly, the findings of this study should be interpreted as reflecting group-level trends rather than individual-level effects.

It should be noted that analyses based on aggregated cell means may influence the interpretation of variability and effect sizes. Specifically, averaging reduces within-cell variance, such that the resulting estimates primarily reflect between-cell differences rather than within-cell variability. Consequently, effect sizes may appear larger, although they may also be attenuated if individual-level variability is averaged out. Therefore, the findings should be interpreted with caution, particularly when comparing them with results derived from individual-level analyses. Data analysis was conducted using IBM SPSS version 24. Correlation analysis was used to address RQ2, regression analysis was employed to examine the relationship between familiarity and nostalgia, and analysis of variance (ANOVA) was used to test differences across age groups corresponding to RQ3.

## 3 Results

**Figure 1:**
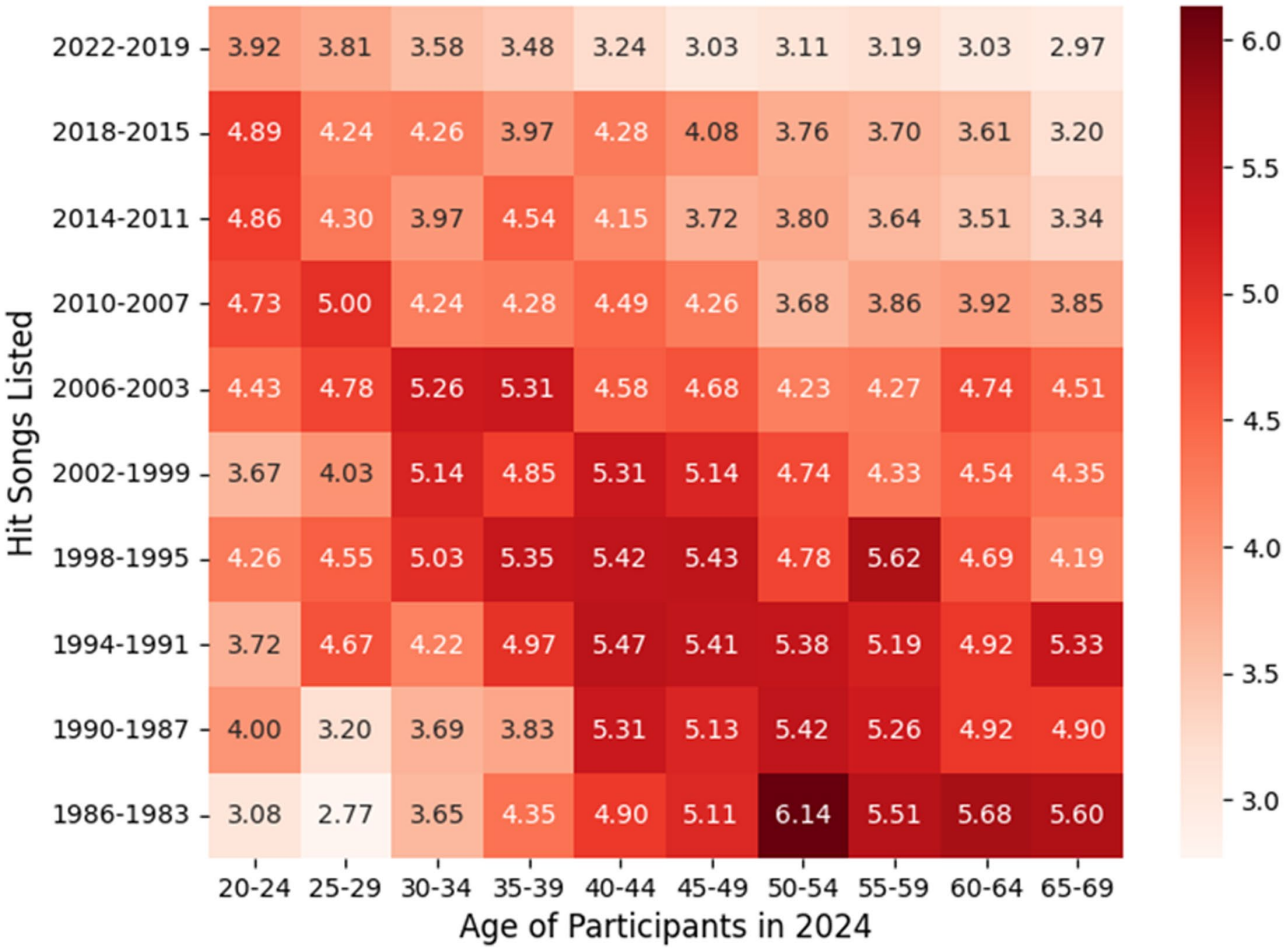
The average nostalgia ratings of participants who looked at the hit lists. The vertical axis represents the years of the hit lists included in the list of songs, and the horizontal axis shows the age of the participants. The numbers in the cells demonstrate the average nostalgia ratings. The values in a single cell represent the average result of approximately 40 participants.

Average nostalgia is shown in Figure 1. Most nostalgia was evoked when participants aged 50–54 years looked at the hit lists from 1983 to 1986 (i.e., between 38 and 41 years before the survey). In general, the value increased from the upper left to the lower right. For example, the hit lists from 2015 to 2018 (i.e., between 6 and 9 years ago) were rated as the most nostalgic by participants aged 20–24 years, while the hit lists from 1995 to 1998 (i.e., between 26 and 29 years ago) were rated as the most nostalgic by participants aged 45–49 years. Therefore, as people age, the hit songs that they feel nostalgic about also become older. In addition, participants generally felt that hit songs that were popular when they were approximately 20 years old were the most nostalgic.

**Figure 2:**
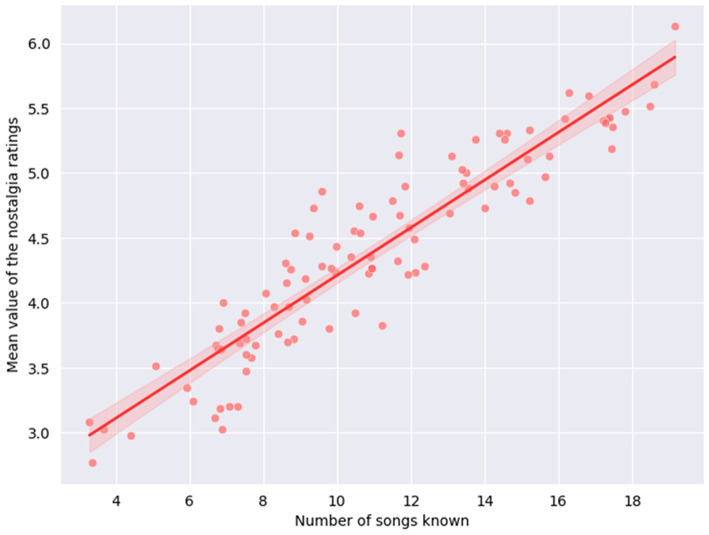
Relationship between the participants’ ratings of nostalgia and the number of songs on the list that they indicated they knew. The rated values, regression line, and 95% confidence intervals are shown. The horizontal axis indicates the number of songs on the list that participants indicated they knew. The vertical axis indicates nostalgia ratings.

The correlation coefficients between the responses to the songs listed on the hit lists, nostalgia, nostalgia function, mood change, and Gold-MSI results are shown in Table 1. It was observed that more nostalgia was evoked if there were more songs that the participants had heard before, regardless of whether they liked them. Additionally, there was a significant positive correlation between the number of favorite songs and the function of nostalgia, with self-continuity displaying a particularly strong correlation. However, there was no relationship between the ratio of songs disliked and nostalgia function. Additionally, there was no association between the responses to the hit lists and changes in mood or Gold-MSI scores. Figure 2 displays the relationship between the number of songs that participants indicated they knew, that is, familiarity, and the nostalgia rating. Linear regression analysis was adopted to model the relationship between familiarity and nostalgia, as linear and quadratic models showed comparable explanatory power. The results of the regression analysis showed that the *R*^2^ values for the linear and quadratic regressions were similar (linear: 0.857, quadratic: 0.864), and a linear regression was adopted based on the distribution of the raw data (*F*(1,98) = 588.0, *p* < 0.001, adjusted *R*^2^ = 0.856, *B* = 0.184, *Const.* = 2.375). The more songs the participants knew from the hit lists, the more monotonous the increase in nostalgia. In other words, if the participants knew one more song, their nostalgia rating increased by 0.18.

**Table 1:** Correlation coefficient between the ratio of songs known in the hit lists and nostalgia, Expanded Function of Nostalgia Scale, mood change, and active engagement factor of Gold-MSI.

	Known	Liked	Disliked	Nostalgia
Nostalgia	0.83**	0.77**	0.68**	-
Function of nostalgia				
Social connectedness	0.21*	0.25*	0.07	0.23*
Self-continuity	0.41**	0.47**	0.16	0.43**
Meaning in life	0.18	0.23*	0.04	0.25*
Self-clarity	0.24*	0.29**	0.07	0.26**
Mood				
Δ Valence	0.13	0.15	0.07	0.12
Δ Arousal	−0.04	−0.01	−0.09	−0.07
Gold-MSI				
Active engagement	−0.05	−0.12	0.10	−0.01

** *p* < 0.01, * *p* < 0.05.

‘Liked’ refers to the ratio of songs in the hit lists that were answered as ‘I have heard of and like the song.’ ‘Disliked’ refers to the ratio of songs in the hit lists that were answered as ‘I have heard of and dislike the song.’ ‘Known’ refers to the percentage of the sum of these two.

**Figure 3:**
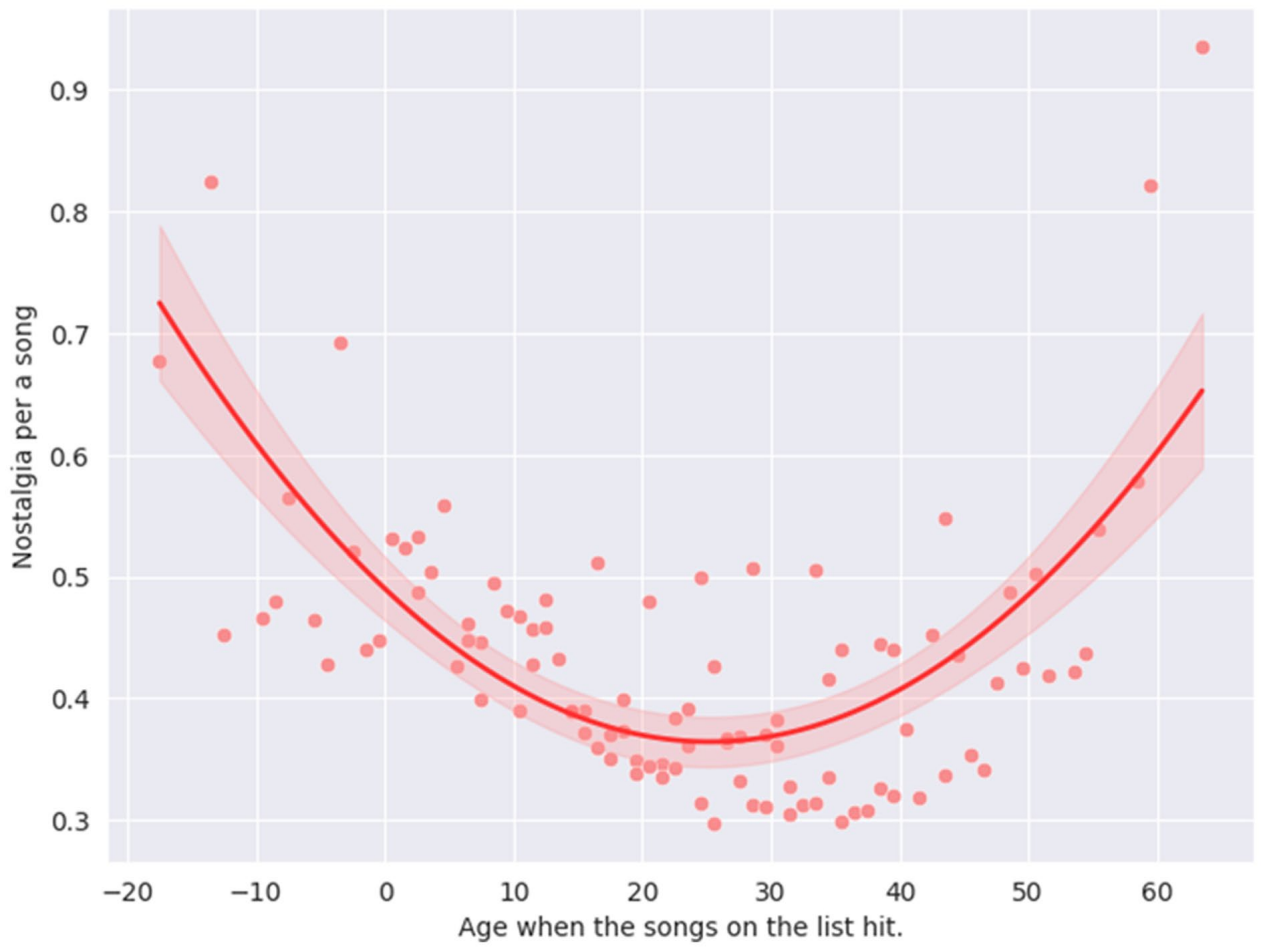
The relationship between the participant’s age at the time the song became a hit and the nostalgia per song. The rated values, regression curve, and 95% confidence intervals are shown. The horizontal axis represents how old the participants were when the songs on the list became hits. Owing to a range of lists and ages, an average value was calculated for each. The vertical axis represents the nostalgia rating divided by the number of songs known.

The relationship between age and nostalgia was calculated by comparing the age of participants when the songs presented to them became hits with the nostalgia felt for each song (Figure 3). The lowest value on the horizontal axis of Figure 3 indicates the response of participants aged 20–24 years when they viewed the hit list between 1983 and 1986, and the highest value was the reaction of participants aged 65–69 years when they looked at the hit list between 2019 and 2022. As the linear regression was not significant, a quadratic regression analysis was conducted (*F*(2,97) = 53.3, *p* < 0.001, adjusted *R*^2^ = 0.514, *β*_2_ = 1.698, *β* = −1.446, *Const.* = 0.458). Consequently, the nostalgia felt for each song was at its lowest around the age of 20. Meanwhile, nostalgia felt for songs that were popular before the participants were born was greater for young participants, and nostalgia felt for songs that were familiar recently was greater for middle-aged participants.

**Figure 4:**
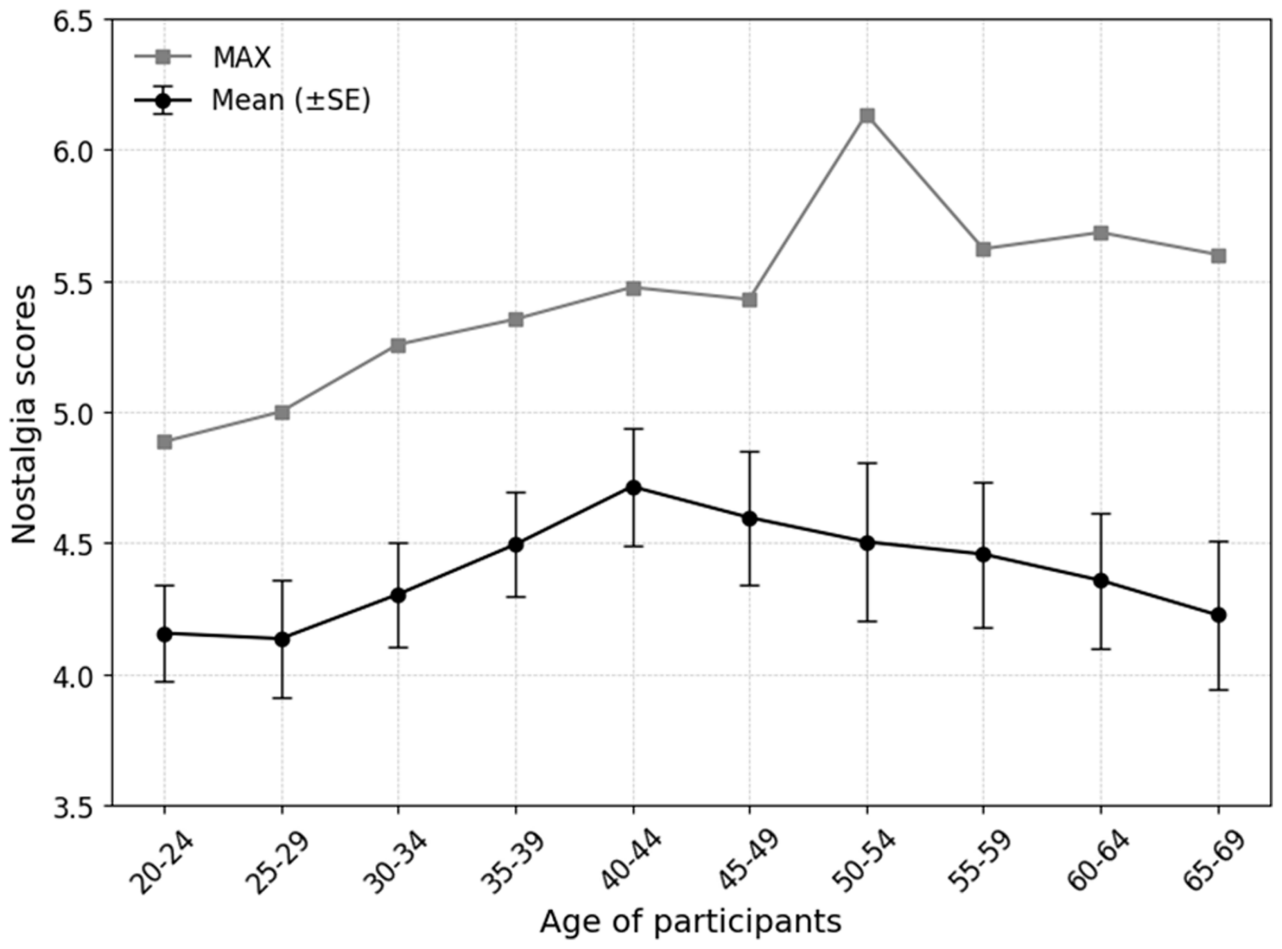
Average and maximum nostalgia scores for each age group. The horizontal axis represents the age group. The vertical axis represents the nostalgia score.

The average and maximum nostalgia scores for each age group are shown in Figure 4. The results of the analysis of variance indicated no significant difference in the average nostalgia scores for the different age groups [*F*(9, 90) = 0.623, *p* = 0.77, *η_p_*^2^ = 0.059]. Although this study presented hit lists for various age groups, no significant differences in nostalgia according to age were observed.

**Figure 5:**
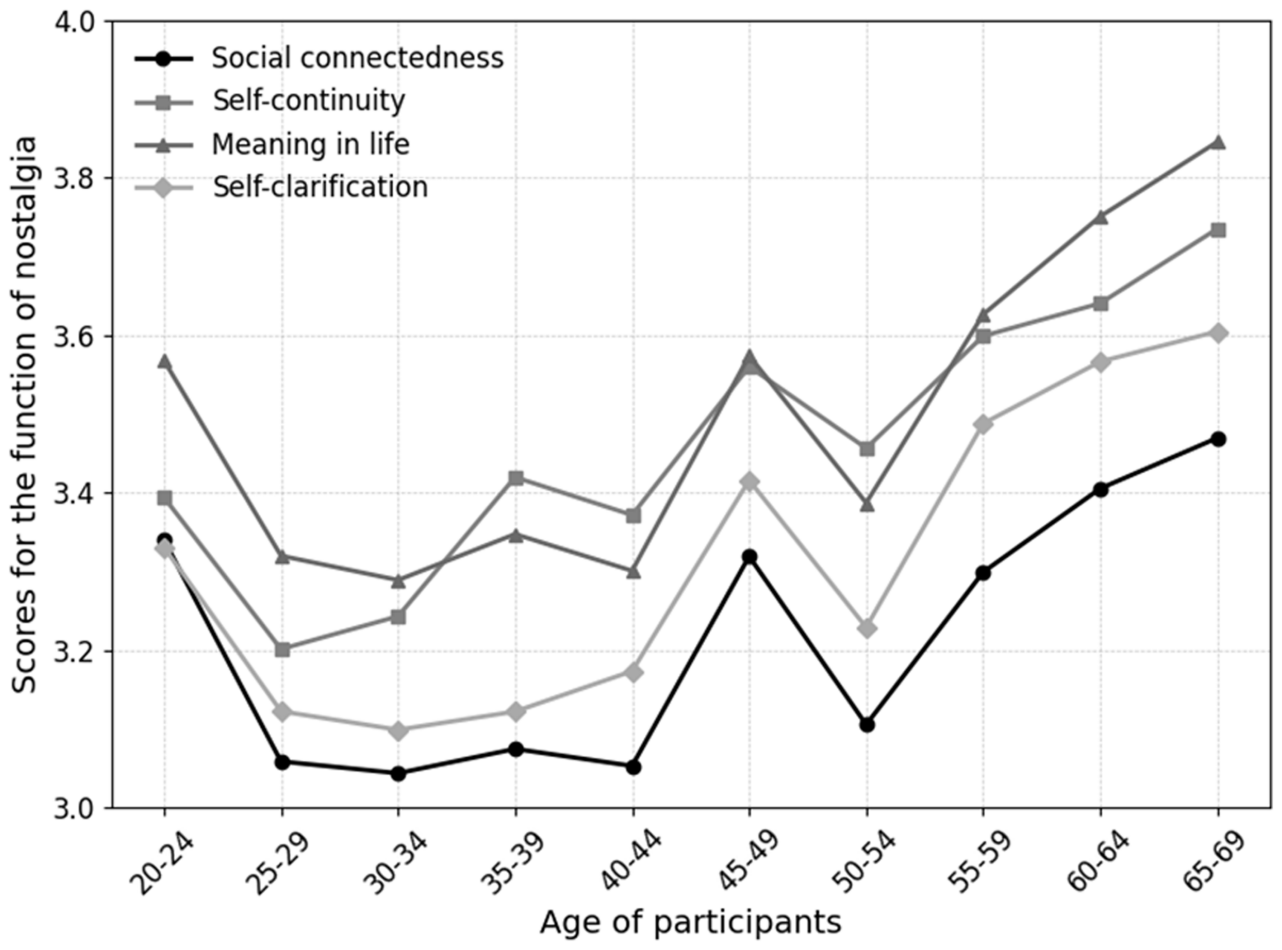
The scores for the function of nostalgia in each age group. The horizontal axis represents the age group. The vertical axis represents the rating for the function of nostalgia.

The average values for the nostalgia function for each age group are shown in Figure 5. A similar tendency was observed for all factors; that is, the values were high in the early 20s, low from the late 20s to early 40s, and increased thereafter.

## 4 Discussion

This study demonstrates that nostalgia can be evoked by informational music cues, such as hit lists presented as text, without requiring auditory music stimuli. The main findings of this study were as follows:

1. Nostalgia was evoked by viewing hit lists alone, without actually listening to the music.
2. The more songs one knew from the hit lists, the more nostalgia and nostalgia functions were evoked. In particular, the correlation with the self-continuity factor was high.
3. The nostalgia evoked by the presentation of the hit lists and its functions were not related to changes in mood or musical experience.

While prior research predominantly focused on nostalgia induced by music listening, the present study demonstrates that informational music cues alone (e.g., song titles and artist names) are sufficient to evoke nostalgia, highlighting a previously underexplored pathway through which nostalgia can be triggered in everyday contexts.

### 4.1 Nostalgia evoked by hit lists

In this study, music evoked nostalgia simply through short text information contained in the hit lists, whereas prior studies have predominantly focused on the induction of nostalgia by actually listening to music (Sedikides et al., 2022). Nostalgia can be evoked by information from various modalities, such as visual (images), auditory (music), and olfactory information (Wildschut et al., 2006). This study validated that short textual titles and names of the musicians were sufficient in evoking nostalgia. This finding suggests that nostalgia is not solely dependent on sensory stimuli but can also be elicited through the recognition of meaningful information. Song titles are not merely strings of characters; they serve as culturally shared symbols and function as part of a semantic network linked to individual autobiographical memories and social experiences. Consequently, the presentation of hit lists is thought to have efficiently activated memories associated with specific past periods or life stages (Baird et al., 2018; Belfi et al., 2015; Cuddy et al., 2015; Jakubowski and Ghosh, 2019; Jakubowski et al., 2020; Jakubowski et al., 2023; O’Shea et al., 2025; Tanberg et al., 2025), thereby inducing nostalgia.

Furthermore, when the participants looked at the hit lists from their 20s, greater nostalgia was reported. This tendency is consistent with the “reminiscence bump” observed in previous research (Jakubowski et al., 2020), and characteristics was observed in other modalities. The results support previous findings that autobiographical memories are triggered by song titles (Jakubowski et al., 2020). Furthermore, in the context of various marketing elements used in advertisements, Kusumi et al. (2010) indicated that music evoked the most nostalgia, whereas product names evoked the least. As their research was based on open-ended responses, the participants did not actually listen to the music, which is in accordance with the present results that nostalgia can be evoked by imagining songs without actually listening to the music.

One possible explanation for this result is that participants recalled music. Although the participants were presented with song titles and performers as textual information in this study, they did not simply respond to textual information but also imagined the hit songs acoustically (if they knew the song) and responded considering that information. The same neural network is used both when listening to music in one’s imagination (musical imagery) and when actually listening to music (Zatorre et al., 1996). Hence, one possible explanation is that participants may have spontaneously engaged in musical imagery; however, this interpretation remains speculative, as imagery was not directly measured in the present study. In this sense, musical imagery should be considered a plausible underlying mechanism rather than a directly observed process in the present study. As listening to music can evoke feelings of nostalgia (Krumhansl and Zupnick, 2013), the hit lists that evoked musical imagery provided feelings of nostalgia similar to those experienced when listening to music. This result is consistent with the finding that music is a trigger for the induction of nostalgia (Barrett et al., 2010; Janata, 2009; Routledge et al., 2011; Sedikides et al., 2022). At the same time, alternative mechanisms may also contribute to this effect. For example, the presentation of song titles may activate semantic memory related to culturally shared knowledge, or prime socially shared memories associated with specific periods. These processes may operate either alongside musical imagery or independently of it.

### 4.2 More known songs evoke more nostalgia

Notably, the more songs participants knew, regardless of whether they liked them, the more nostalgia was evoked. The association between the number of known songs and the intensity of nostalgia suggests that nostalgia is not a phenomenon in response to a single stimulus; rather, it may be amplified through the cumulative presentation of multiple memory cues. If hit lists evoke only a certain degree of nostalgia (i.e., whether they are nostalgic), this result may seem strange. This is because regardless of the number of known songs, they are rated as nostalgic. However, the results showed a directly proportional relationship between the number of known songs and how nostalgic they were. These results can be interpreted from the following perspectives. First, the fact that there were many songs the participants knew provided them with many clues about that era, which led to a higher nostalgia rating. Nostalgia is caused by an awareness of the past. Second, the hit lists used in this study covered four consecutive years. Therefore, the fact that the participants knew many songs meant that they had more clues about the period. Hit lists evoke autobiographical memories (Jakubowski et al., 2020). This suggests that many well-known songs evoke more nostalgia by inducing more autobiographical memories. Alternatively, a large number of memory cues may make memories of that time clearer, which, in turn, evokes more nostalgia.

Another possibility is that known songs synergistically provide nostalgia. It is possible that nostalgia was evoked by a certain known song and musician on the hit lists and that nostalgia was further promoted by another known song and musician. Actively amplifying a positive experience leads to greater happiness (Jose et al., 2012). Similarly, in this study, nostalgia may have been maintained by a nostalgic mood. Eudaimonia (a better life), a positive emotion, is associated with the function of nostalgia (Layous et al., 2022). In this study, the fact that social connections (connections with other people) and meaning of life (the value and purpose of living) were also evoked may support this finding. Indeed, a positive correlation was found between the number of favorite songs that induced positive emotions and the nostalgia function of known songs. However, no correlation was found for songs that were disliked. This finding suggests that the presentation of favorite songs evokes nostalgia and positive emotions that promote the function of nostalgia.

However, the weight of nostalgia for each song was not always the same. Although older people do not know much about recent songs and young people do not know much about songs before their birth, if individuals know songs, such songs are likely to evoke nostalgia. This result suggests that when people know songs from a time that they do not directly associate with, such as during adolescence, songs are more likely to be associated with a special experience or memory. Particularly, for songs that charted before birth, the music an individual’s parents listened to may have impacted their children (Krumhansl and Zupnick, 2013).

### 4.3 The list of songs functioned as a trigger to connect the past and present

The strongest association was observed between self-continuity and nostalgia, suggesting that informational music cues may support the psychological linkage between past and present selves through symbolic cultural references. The more preferred songs on the hit lists, the greater the nostalgia function evoked. In particular, there is a strong correlation between preferred songs and factors that recognize temporal continuity from the past. Consequently, the hit lists could act as a trigger to make the past and present feel like a single thing. This type of self-continuity (Sedikides and Wildschut, 2022), a sense of coherence, is essential for psychological health (Sedikides et al., 2023). However, previous research (Kusumi, 2021) has shown that continuity of time factors do not lead to life satisfaction; rather, social connectedness and self-definition are related to life satisfaction. In this study, combined with the fact that nostalgia was not correlated with mood changes, looking at the hit lists did not directly lead to mood improvement. This may be because the hit lists in this study differed from the images of Japan’s original scenery presented in a previous study. In other words, as Figure 1 suggests, as the list of songs is associated with nostalgia for a particular period of one’s life, there may be an awareness of a temporal sequence that links the past and the present.

### 4.4 Social bonds generated by hit lists

The present results suggest that nostalgia induced by music can generate social bonds, consistent with previous findings (Kusumi, 2021). Given that hit lists represent culturally shared artifacts, nostalgia evoked by such lists may facilitate a sense of collective memory and social connectedness. Music can produce an effect that deepens social bonds (Dunbar et al., 2012). The present results added a new perspective to the recent surge in research on social bonds by including a research design that involves looking at the song titles and names of musicians. These results could also be explained by the familiarity of the hit songs. Research on synchrony has shown that when music is more familiar, social closeness increases regardless of movement synchrony (Stupacher et al., 2020). The songs listed in the hit lists may have been heard by many people and can be considered a shared experience for each generation. Sharing collective memories with others may promote communication and social bonding. However, the evidence for social bonds in the present study is relatively indirect. Although a clear association was observed for self-continuity, the relationship with social bonds was weak and should therefore be interpreted with caution. Therefore, as the present study focused on psychological responses rather than behavioral indicators of social interaction or bonding, these interpretations should be understood as preliminary.

### 4.5 Nostalgia and changes in mood

The absence of significant mood change suggests that informational nostalgia does not necessarily translate into immediate affective modulation, in contrast to findings from music listening studies (Barrett et al., 2010). Nostalgia has been reported to be a primarily positive emotion (Leunissen et al., 2021; Wildschut et al., 2006), and to provide a sense of happiness in one’s life (Zhou et al., 2022). Thus, it was presumed that there would be a positive correlation between nostalgia and positive emotions. However, the results revealed no significant relationship. One possible explanation for this finding concerns the nature of the mood measure used in the present study. The Two-Dimensional Mood Scale was primarily employed to capture short-term affective states (e.g., valence and arousal) in this study and may therefore be less sensitive to the more meaning-oriented or existential aspects of nostalgia. Nostalgia has been suggested to involve processes related to self-continuity, meaning in life, and social connectedness (Sedikides et al., 2016; Sedikides and Wildschut, 2022), which may not be fully reflected in transient mood changes. In this sense, the absence of a significant relationship between nostalgia and mood change does not necessarily contradict previous findings showing positive emotional effects of nostalgia but may instead reflect differences in the level of emotional processes being assessed. Another possible reason for this is that actual music was not presented. Listening to music can cause changes in emotion (Juslin and Laukka, 2004). Therefore, reading textual information about music may not affect emotion.

Nostalgia also has ambivalent characteristics as it is considered to be bittersweet (Holak and Havlena, 1998; Sedikides and Wildschut, 2022; Sedikides and Wildschut, 2018). Homesickness is a negative emotion associated with nostalgia (Newman et al., 2020) and, considering the present result that nostalgic songs are biased towards adolescence, there may be an aspect of a negative emotional response to a ‘past that cannot be returned to.’ Owing to this mixture of negative and positive emotions, no clear link between nostalgia and emotional change was observed. Considering the argument that nostalgia induced by music is not just a benefit (Sedikides et al., 2022), this study suggests the possibility that a simple formula for mood enhancement with nostalgic music is not always applicable.

### 4.6 Limitations and future research

Although this study revealed that hit lists evoke nostalgia and nostalgia-related functions, the mechanisms underlying this phenomenon remain unclear. In other words, the question remains: Did the participants recall autobiographical memories from the hit lists, or did the participants recall songs themselves and become influenced by their impressions of the songs? Alternatively, was this phenomenon a combination of these factors? To clarify this issue, studies using various types of stimuli, such as auditory stimuli, would be effective. Further investigations should also clarify the various psychological responses induced by nostalgia, such as the functions of nostalgia, in addition to nostalgia itself. A further limitation concerns the role of mood. Although mood was included as a variable in the present study, no significant relationship was observed between nostalgia and changes in mood. This suggests that the type of nostalgia examined here—evoked by informational music cues—should not be interpreted as necessarily involving immediate changes in affective state. Rather, the findings indicate that this form of nostalgia may be more closely related to meaning-oriented psychological processes, such as self-continuity and social connectedness, than to short-term mood fluctuations.

### 4.7 Conclusion

The present study demonstrates that nostalgia can be elicited by informational music cues, such as hit lists presented as textual information, even in the absence of music listening. Short text information can lead to a psychological response of nostalgia, contribute to the recognition of the meaning of life, and cultivate social bonds. Therefore, a large-scale dataset of Japanese hit songs provides novel cross-cultural evidence on how culturally shared musical information functions as a cue for nostalgia. These findings suggest that symbolic cultural artifacts related to music can support autobiographical meaning-making and a sense of self-continuity. In particular, social bonds as a function of nostalgia may be useful for addressing the pervasive issue of loneliness in recent years (Abeyta et al., 2020; Kawase and Eguchi, 2025). From another perspective, a streaming service recommendation system may potentially provide a more satisfactory listening experience by presenting titles of songs and names of musicians to specific users in advance. The effect of music, which is an important trigger for nostalgia, may be achieved to some extent with textual information alone, and can be applied in situations where playing music is challenging. Future research that incorporates the present findings is expected to examine actual listening behavior, consumption behavior, and social interaction. Overall, hit lists are likely to have many socially useful applications. Future studies should directly compare nostalgia evoked by informational cues and music listening and examine the distinct psychological mechanisms underlying these experiences.

## Data Availability

The original contributions presented in the study are included in the article/supplementary material, further inquiries can be directed to the corresponding author.
